# One-Dimensional (NH=CINH_3_)_3_PbI_5_ Perovskite for Ultralow Power Consumption Resistive Memory

**DOI:** 10.34133/2021/9760729

**Published:** 2021-10-08

**Authors:** Xuefen Song, Hao Yin, Qing Chang, Yuchi Qian, Chongguang Lyu, Huihua Min, Xinrong Zong, Chao Liu, Yinyu Fang, Zhengchun Cheng, Tianshi Qin, Wei Huang, Lin Wang

**Affiliations:** ^1^ Key Laboratory of Flexible Electronics (KLOFE) & Institute of Advanced Materials (IAM), Nanjing Tech University (Nanjing Tech), 30 South Puzhu Road, Nanjing 211816, China; ^2^ Electron Microscope Laboratory, Nanjing Forestry University, Nanjing 210037, China; ^3^ MIIT Key Laboratory of Flexible Electronics (KLoFE), Shaanxi Key Laboratory of Flexible Electronics (KLoFE), Xi’an Key Laboratory of Flexible Electronics (KLoFE), Xi’an Key Laboratory of Biomedical Materials & Engineering, Xi’an Institute of Flexible Electronics, Institute of Flexible Electronics (IFE), Northwestern Polytechnical University, Xi’an, 710072 Shaanxi, China

## Abstract

Organic-inorganic hybrid perovskites (OIHPs) have proven to be promising active layers for nonvolatile memories because of their rich abundance in earth, mobile ions, and adjustable dimensions. However, there is a lack of investigation on controllable fabrication and storage properties of one-dimensional (1D) OIHPs. Here, the growth of 1D (NH=CINH_3_)_3_PbI_5_ ((IFA)_3_PbI_5_) perovskite and related resistive memory properties are reported. The solution-processed 1D (IFA)_3_PbI_5_ crystals are of well-defined monoclinic crystal phase and needle-like shape with the length of about 6 mm. They exhibit a wide bandgap of 3 eV and a high decomposition temperature of 206°C. Moreover, the (IFA)_3_PbI_5_ films with good uniformity and crystallization were obtained using a dual solvent of N,N-dimethylformamide (DMF) and dimethyl sulfoxide (DMSO). To study the intrinsic electric properties of this anisotropic material, we constructed the simplest memory cell composed of only Au/(IFA)_3_PbI_5_/ITO, contributing to a high-compacted device with a crossbar array device configuration. The resistive random access memory (ReRAM) devices exhibit bipolar current-voltage (*I-V*) hysteresis characteristics, showing a record-low power consumption of ~0.2 mW among all OIHP-based memristors. Moreover, our devices own the lowest power consumption and “set” voltage (0.2 V) among the simplest perovskite-based memory devices (inorganic ones are also included), which are no need to require double metal electrodes or any additional insulating layer. They also demonstrate repeatable resistance switching behaviour and excellent retention time. We envision that 1D OIHPs can enrich the low-dimensional hybrid perovskite library and bring new functions to low-power information devices in the fields of memory and other electronics applications.

## 1. Introduction

Organic-inorganic hybrid perovskites (OIHPs), which exhibit the advantage of tunable bandgap [1], high bipolar carrier mobility [2], long carrier diffusion length [3], and structural diversity [4], become one of the most promising materials in physical electronic applications [5], not just photovoltaic applications [6–8] and light-emitting diodes [9–11] (LEDs). The inherent ion migration of OIHPs easily causes current-voltage hysteresis, which leads to the instability and inefficiency of photovoltaic devices [12]. However, the sustainable current hysteresis phenomenon is a booming chance for applications in logic circuit [13], data storage [14], and resistance switching [15–17]. In recent years, resistive random access memory (ReRAM) devices based on OIHPs possess high integration density [18], good scaling capability [19], and multilevel information storage [20], which make them a promising candidate for the next-generation computing system. These excellent performances of ReRAM devices are mainly affected by the material structure of synthesized OIHPs [21]. Apart from fine-tuning the component and thickness of OIHPs, their dimensionality modulation also helps to optimize the performances of ReRAM devices.

In recent years, three-dimensional (3D) perovskites (such as CH_3_NH_3_PbI_3_ [22] and CsPbBr_3_ [23]) have been applied to resistive random access memory (ReRAM) devices, which reflect reproducible resistive switching behaviour and flexible applications. The ReRAM devices based on two-dimensional (2D) Ruddlesden-Popper (RP) perovskites have better environmental stability and endurance properties owning to the protection of large organic cations on the surface [24]. The on/off ratio of memristor based on 2D OIHPs was dramatically increased due to much higher Schottky barrier between the metal electrode and active layer [25]. Moreover, compared with 3D OIHPs, 2D functional layer is anisotropic which significantly reduces the formation energy barrier of conductive filaments along the defect channels [26]. However, the research on ReRAM devices based on single 1D OIHPs is still in its infancy, with none study reported, which is a direct and effective way to determine the intrinsic performance characteristics and applicable functional devices of new materials.

Here, we develop a solution-processed method to obtain high-quality, needle-like shape, and large-size (∼6 mm in length) 1D (NH=CINH_3_)_3_PbI_5_ ((IFA)_3_PbI_5_) crystals of a monoclinic crystal phase.

(1)N≡CNH2+2HI⟶NH=CINH3I

(2)3NH=CINH3I+PbI2⟶NH=CINH33PbI5

The well-crystallized (IFA)_3_PbI_5_ owns a high decomposition temperature of ~206°C and a wide bandgap of ~3.0 eV, as compared with that of OIHPs in 2D and 3D. To explore the resistive switching characteristics of 1D (IFA)_3_PbI_5_, we prepared (IFA)_3_PbI_5_ films on ITO-coated glasses using a mixed solvent of N,N-dimethylformamide (DMF) and dimethyl sulfoxide (DMSO). The dual-solvent method helped us overcome the strong orientation and poor film-forming problem of low-dimensional OIHPs. Scanning electron microscope (SEM) and atomic force microscopy (AFM) analyses confirm that the large-area thin films possess high-quality crystallization and controllable uniformity, being well controlled by the molar ratio of DMF and DMSO. Then, we successfully manufactured ReRAM devices based on the simplest sandwich structure composed of Au/(IFA)_3_PbI_5_/ITO. The structure is no need to require any additional insulating layer or double metal electrodes, in which (IFA)_3_PbI_5_ film acts as the only insulating layer, benefitting facile device fabrication and intrinsic property study. In particular, the devices show the record-low power consumption of 0.2 mW among all OIHP-based memristors. Moreover, our devices have the lowest power consumption and “set” voltage (0.2 V) among all perovskite-based memristors that do not require double metal electrodes or any additional insulating layer. The fabricated 1D OIHPs provide a new opportunity for resistive switching applications, indicating that the material design of low-dimensional perovskites is of great potential in multifunctional electronic applications.

## 2. Results and Discussion

**Figure 1:**
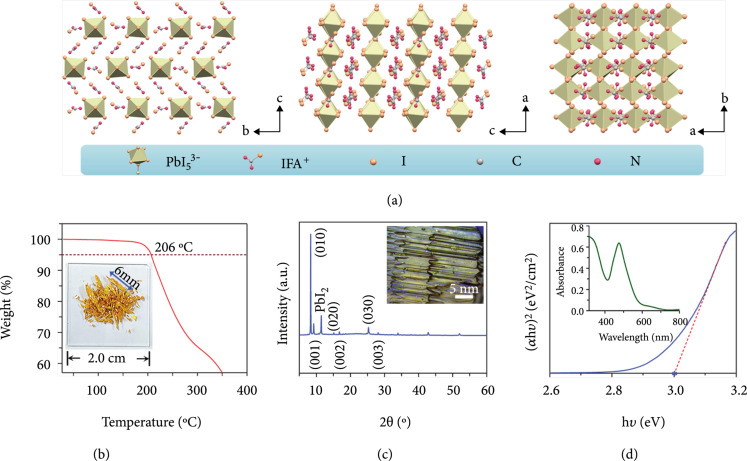
Architecture and characterization of one-dimensional (IFA)_3_PbI_5_ crystal. (a) Schematic illustrations of the crystal structures along the axes of a, b, and c, respectively. (b) Thermal stability of (IFA)_3_PbI_5_ perovskite. The inset shows the needle-like shape of 1D crystal with a size of about 6 mm. (c) XRD spectrum of (IFA)_3_PbI_5_ film spin-coated on an ITO substrate. Inset: microscope picture of (IFA)_3_PbI_5_ perovskite. (d) The bandgap of (IFA)_3_PbI_5_ is about 3 eV, as determined by absorption spectrum (inset).

(IFA)_3_PbI_5_ is a new OIHP with 1D chain-shaped crystal structure, as characterized by X-ray single-crystal diffractometer. 1D (IFA)_3_PbI_5_ is a subclass of ABX_3_ materials in which the A-site is replaced by carbamimidic iodide cations of IFA^+^. The organic linker intersects and cuts along two special crystallographic planes of (110) and (010), breaking space constraints of conventional perovskites. Our 1D perovskite is formed by a large organic cation of IFA^+^ alternating with a 1D inorganic chain of PbI_5_^3-^. As shown in Figure 1(a), structural illustrations of 1D perovskite on different planes show the case with five I atoms surrounding each Pb atom, in which one I atom in-chain is shared by two octahedrons, forming a 1D shape of PbI_5_^3-^. The negative charges are compensated by the large organic cation IFA^+^ that caps the surface of 1D chains. The production of IFA^+^ units is a precondition for forming the chain of 1D (IFA)_3_PbI_5_ perovskite. This production process requires breaking the carbon-nitrogen triple bond of cyanamide and then forming a carbon-iodine bond by adding iodine, thereby breaking the coulomb and hydrogen bonds in the 3D perovskite structure.

The crystal products display a needle-like shape (the inset of Figure 1(b)) and a large size with a representative length of about 6 mm. This crystal demonstrates good thermal stability with 5% weight loss at 206°C (Figure 1(b)) by using thermogravimetric analysis. The crystalline orientations could be detected by X-ray diffraction (Figure 1(c)), indicating a strong 2*θ* peaks at about 8.3° may be an XRD characteristic peak of 1D hybrid perovskite that is similar to the low-dimensional diffraction peaks of 2D OIHPs [27]. As shown in Table S1, all the XRD peaks are indexed to a monoclinic perovskite phase (p2(1)/c space group) with a=6.430 Å, b=20.129 Å, and c=18.793 Å. Besides, all the XRD peaks exhibit regular diffraction orientations, indicating that the prepared (IFA)_3_PbI_5_ film is highly crystalline, consistent with the clear and regular 1D orientations of the bulk crystal observed using a microscope (inset of Figure 1(c)). We further studied the bandgap of 1D (IFA)_3_PbI_5_ by absorption spectrum, as displayed in the inset of Figure 1(d). The bandgap of (IFA)_3_PbI_5_ is about 3.0 eV, being wider than that of the perovskites with other dimensions [28]. We infer that the dimension could be an important parameter to control the band structure of hybrid perovskite.

**Figure 2:**
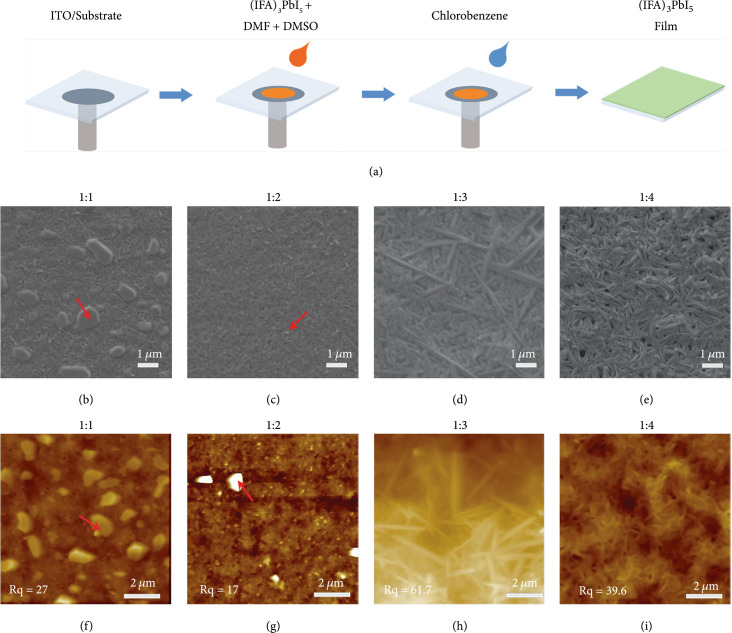
Fabrication and characterization of (IFA)_3_PbI_5_ films. (a) Schematic illustration of the fabrication process of (IFA)_3_PbI_5_ film on an ITO substrate. Representative SEM images of the (IFA)_3_PbI_5_ films prepared by the molar ratios of (IFA)_3_PbI_5_ to DMSO of (b) 1 : 1, (c) 1 : 2, (d) 1 : 3, and (e) 1 : 4; corresponding AFM images are shown in (f–i), respectively. The red arrows point to the agglomerates that have not been fully dissolved.

Fabricating high-quality and uniform films is an effective measure to scale up the size and enhance the properties of OIHP-based devices. A cosolvent of DMF and DMSO was applied to prepare films of 1D (IFA)_3_PbI_5_, because the strong coordinate effect of DMSO helps to uniform crystal growth rates and promote crystallization of (IFA)_3_PbI_5_ in solvents [29]. We fixed the DMF volume (160 *μ*L) and (IFA)_3_PbI_5_ (168 mg) and tuned the molar ratios of (IFA)_3_PbI_5_ to DMSO to 1 : 1, 1 : 2, 1 : 3, 1 : 4, and 1 : 5, respectively, to find the optimized conditions for (IFA)_3_PbI_5_ films. In a glove box with nitrogen atmosphere, (IFA)_3_PbI_5_ films were successfully fabricated on ITO-coated glasses (Figure 2(a)) by adding a trace of antisolvent (chlorobenzene) at the last five seconds of spin-coating for accelerating the film crystallization. We examined the products of this reaction by scanning electron microscope (SEM) and atomic force microscopy (AFM). Our films of 1D (IFA)_3_PbI_5_ show the same clear 1D needle-like shapes as the bulk crystals, which is different from the fuzzy surface morphology of other 1D perovskite films [30]. As shown in Figures 2(b)–2(e) and Figure S1(a), SEM images identify the full covering of needle-like perovskites on ITO substrates. The aggregates that have not been completely dissolved are indicated by red arrows (Figures 2(b) and 2(c)), describing that the solubility of (IFA)_3_PbI_5_ is well controlled by the dose of DMSO. The obvious pinholes are observed on the film surface, as shown by the yellow arrow in Figure S1(a), suggesting poor film is prepared using high dose of DMSO. As the molar ratio of (IFA)_3_PbI_5_ to DMSO increases from 1 : 1 to 1 : 5, the average size of the needle shape increases from ~286 nm to ~895 nm (Figure S2). The above data suggest that crystallization of (IFA)_3_PbI_5_ film can be precisely controlled by the dual-solvent method. AFM images in Figures 2(f)–2(i) and Figure S1(b) further verify the good control effect of DMSO on crystal solubility and further reveal that DMSO produces a good effect in film uniformity that the large-size needle shapes have a relatively small roughness ($R_{q}=\sim 39$ nm, Figure 2(i)). These data clearly illustrate the 1D features of (IFA)_3_PbI_5_, in which the films present relatively lower roughness, larger crystal grains, and higher quality when the molar ratio of 1D crystal to DMSO is of 1 : 4 during film production (Figure S2(d)).

**Figure 3:**
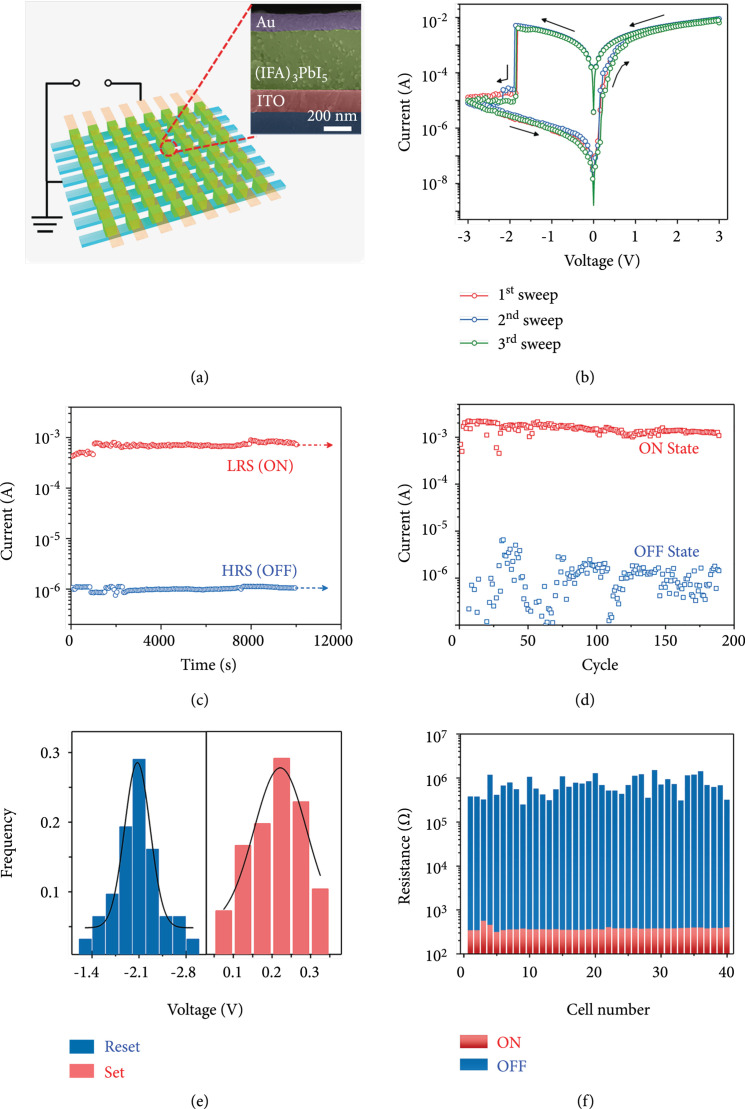
Resistive switching properties of (IFA)_3_PbI_5_-based memristors. (a) Schematic and circuit diagram of a ReRAM device with acrossbar array configuration. The measurement unit cell with a sandwich structure composed of Au/(IFA)_3_PbI_5_/ITO is indicated by a redcircle. Inset: SEM cross-section image of a memristor cell. (b) Typical *I-V* curves with three sweeps of an Au/(IFA)_3_PbI_5_/ITO memory device. DC switching of (c) the retention time test and (d) nearly 200 consecutive cycles; the currents were read at -0.5 V. (e) Histogram statistics of set/reset voltage distributions. (f) The resistance statistics of 40 representative unit cells.

Storage properties are important to evaluate the quality of synthesized films, especially the storage properties of 1D OIHPs that have not been reported. Here, ReRAM devices are constructed based on (IFA)_3_PbI_5_ films with a molar ratio of 1 : 4 of (IFA)_3_PbI_5_ to solvent DMSO. As shown in the inset of Figure 3(a), the thickness of the (IFA)_3_PbI_5_ layer is about 450 nm from the SEM cross-section view of a memristor cell. To research the intrinsic resistive switching properties of (IFA)_3_PbI_5_ perovskite, we fabricated the (IFA)_3_PbI_5_ film without any additional interface into the simplest sandwich structure of Au/(IFA)_3_PbI_5_/ITO, as further conveniently integrated into an 8×8 crossbar array memory (Figure 3(a)). Each unit cell area is 0.01 mm^2^, of which electric properties were tested under ambient conditions. Figure 3(b) shows typical resistive switching (RS) behaviour of an Au/(IFA)_3_PbI_5_/ITO device under the DC voltage sweep sequence of 0 V→3 V→0 V→-3 V→0 V, which exhibits nonvolatile resistive storage (NVRS) behaviour. The overlap of three successively measured *I-V* loops reflects the good reliability and forming-free properties of the device. From the virgin state, the first voltage sweep (as the red curve shown in Figure 3(b)) was performed by applying a positive bias on the Au top electrode, until a positive bias was applied to switch the device to LRS, which is commonly referred to as a “set” process. During this process, the device displays the conversion bias of “set” which is about 0.2 V, possessing an ultralow power consumption of about 2 mW (note that power consumption is equal to LRS current multiplied by set voltage). Then, LRS maintains until a large negative bias voltage was used, and the *I-V* curves are converted to HRS at about -2.1 V, which is known as the “reset” process. The switching characteristics at opposite polarity indicate that the memristor exhibits bipolar switching. The negative conversion voltage is significantly greater than the “set” bias, which is related to the conduction mechanism of the Au/(IFA)_3_PbI_5_/ITO device, as explained below.

We further check the reliability and reproducibility of our devices. With a reading voltage ($V_{\text{read}}$) of -0.5 V, we measured the data retention characteristics of HRS and LRS values and the cycling endurance, as shown in Figures 3(c) and 3(d), respectively. A constant on/off ratio of 10^3^ is maintained for up to 10^4^ s. Both LRS and HRS are relatively stable showing excellent repeatability between HRS and LRS for nearly 200 cycles. Moreover, we statistically calculated these basic characteristics from other 40 unit cells. Figure 3(e) presents the histograms of voltage distribution for the set (red) and reset (blue). The average voltage values of the set and reset are 0.2 and -2.1 V, respectively. Figure 3(f) shows the statistical resistance values of HRS and LRS of these 40 cells, respectively, with a stable value of the on/off ratio around 10^3^.

Table 1 summarizes the performance comparison between our work and other good demonstrations of perovskite-based RS devices reported in recent years [15, 31–37]. The memristors based on 1D (IFA)_3_PbI_5_ in our work own the record-low power consumption among all OIHP-based memristors [15, 31–33]. It is also worth noting that most memristors own low power consumption with the setting of compliance current, normally lower than the current that the device can reach if without setting [31–37]. Also, (IFA)_3_PbI_5_-based memristors work normally without installing the compliance current, which show the robustness of our devices. Moreover, compared to the devices with the simplest device structure, such as Au/CH_3_NH_3_PbI_3_/ITO [32], Au/CH_3_NH_3_PbI_3−*x*_Cl*_x_*/FTO [33], and Al/CsBi_3_I_10_/ITO [37], which do not require double metal electrodes or any additional insulating layer, our devices own the lowest power consumption and “set” voltage (0.2 V). The simplest device structure is advantageous for simplifying device fabrication process and studying material intrinsic properties.

**Table 1:** Comparison of 1D (IFA)_3_PbI_5_-based memristor (our work) with previous works.

Device structure	Power consumption	Set voltage	On/off ratio	Retention time
Our work	**0.2 mW**	**0.2 V**	**10** ^**3**^	**10** ^**4**^
Organic-inorganic hybrid perovskites based devices				
Ag/FAPbI_3_/Pt [31]	0.22 mW	0.22 V	10^5^	$3\times 10^{3}$
Au/CH_3_NH_3_PbI_3_/ITO [32]	0.7 mW	0.7 V	10^2^	10^4^
Ag/CH_3_NH_3_PbI_3_/Pt [15]	0.75 mW	0.15 V	10^6^	$1.1\times 10^{4}$
Au/CH_3_NH_3_PbI_3−*x*_Cl*_x_*/FTO [33]	14.7 W	1.47 V	10^4^	$4.32\times 10^{4}$
Inorganic perovskite - based memory devices				
Au/Cs_3_Bi_2_I_9_/Pt [34]	0.1 mW	0.1 V	10^7^	10^3^
Ag/PMMA/AgBiI_4_/ITO [35]	0.16 mW	0.16 V	10^4^	10^4^
Ag/PMMA/CsPbI_3_/Pt [36]	0.18 mW	0.18 V	10^6^	10^3^
Al/CsBi_3_I_10_/ITO [37]	1.7 mW	1.7 V	10^3^	10^4^

**Figure 4:**
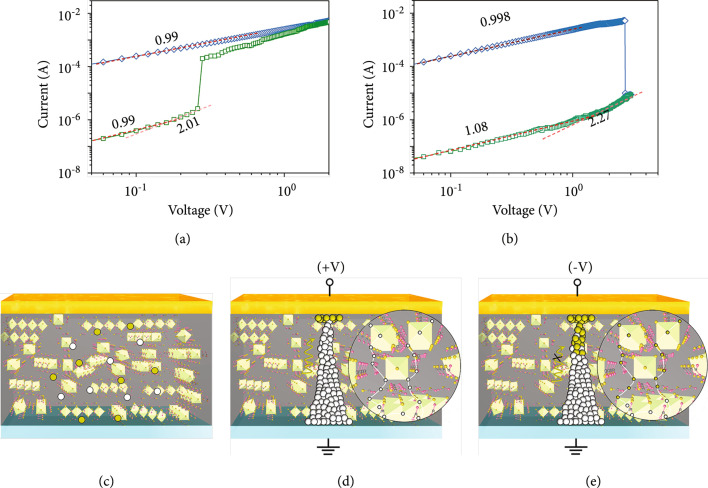
Resistive switching mechanisms. Analysis of Au/(IFA)_3_PbI_5_/ITO on the *logI-logV* curves and their fitting results of (a) “set” and (b) “reset” under voltage sweep. (c–e) Schematic diagrams of the process of switching mechanism, and the iodine ions and iodide vacancies ($V_{I}$’s) are represented by the green and white balls, respectively.

For investigating the conduction mechanism in detail, we plot the *I-V* curves for the set process with a double logarithmic (see Figure 4(a)). The HRS data in the voltage region of 0-0.15 V is fitted well by a linear relationship with a slope of ~0.99, indicating a good Ohmic conduction [38]. This suggests that intrinsic thermally generated free carriers inside the (IFA)_3_PbI_5_ films are predominant over the injected carriers by electrical field. With the positive voltage rising from 0.15 V to 0.2 V, the injected carriers exceed thermally generated carriers that make a trap-controlled space charge limit current (SCLC) behaviour [39] (I-V^2^). When voltage is larger than 0.2 V, all of the traps are filled by charge carriers and the conductive paths are formed in (IFA)_3_PbI_5_ films [40], setting the device from HRS to LRS. For the whole LRS range, the slope of *I-V* curves remains ~0.99, demonstrating an Ohmic conduction mechanism. Moreover, the fitting results of *I-V* curves exhibit the similar mechanism for the reset process, as shown in Figure 4(b), suggesting the reverse formation and rupture of conduction paths.

Inspired by the analysis of electric properties on a logarithmic scale, we further investigate the possible switching mechanism of our 1D (IFA)_3_PbI_5_-based devices. Halide perovskite as an active layer in NVRS is generally regarded as relatively soft ionic solids, which is prone to contain point defects, vacancies, interstitials, cations, and antisite substitutions [41]. Previous work demonstrated that iodide vacancies ($V_{I}$’s) have much lower activation energy (0.1-0.6 eV) compared with other point defects [42]. Therefore, we think $V_{I}$’s conductive filament is very possibly responsible for the conductive mechanism in our 1D (IFA)_3_PbI_5_-based memristors. As shown in Figure 4(c), many thermally generated iodine ions and their corresponding vacancies are randomly distributed in (IFA)_3_PbI_5_ films before applying a voltage bias. As shown in Figure 4(d), when a positive voltage bias is applied to a memristor, $V_{I}$’s conductive filament paths begin to create after the iodine ions and their vacancies migrate toward the opposite directions. The conductive filaments grow along $V_{I}$ defect channels from the bottom electrode to the top electrode, facilitating the injected carriers following trap-to-trap hopping, thereby prompting the memory device from HRS to LRS. Afterwards, in the negative voltage region, the redistribution of iodine ions and their vacancies brings the collapse of $V_{I}$’s channels, and the device was recovered from LRS to HRS (Figure 4(e)).

As a functional layer, 1D (IFA)_3_PbI_5_ owns obvious anisotropic characteristics, which has natural 1D channels for the orderly migration of ions in the dark field, thereby greatly reducing the energy barrier for the formation of conductive paths. Therefore, it is possible that the $V_{I}$’s conductive filaments can easily formed under an extremely low electric field in (IFA)_3_PbI_5_ perovskite with native 1D crystal structure, leading to the device low operating voltage and ultrapower consumption. Meanwhile, the high-density 1D ion channels in the active layer provide convenience for the growth of multiple conductive filaments with robust features. It is difficult to break these robust conductive channels, thus requiring a large negative voltage to convert the device from LRS to HRS.

## 3. Conclusions

In summary, we show the controllable fabrication and storage properties of 1D (IFA)_3_PbI_5_ formed by carbamimidic iodide cations. The large-size crystals with needle-like shapes own good thermal stability and wide bandgap. We also produce (IFA)_3_PbI_5_ films using a dual-solvent method. The morphology structure of the films was readily controlled by the solvent ratio of DMF to DMSO. Our films possess good crystallization and uniformity, allowing the first investigation of resistive switching properties of 1D perovskite. We apply the simplest ITO-based sandwich device structure with a single metal electrode, in which (IFA)_3_PbI_5_ is used as the only insulating layer, to simplify the fabrication process and reveal the intrinsic storage characteristics. The memristors possess bipolar nonvolatile resistive switching behaviour, particularly with a record-low power consumption of about 0.2 mW among all OIHP-based memories. Also, our devices own the lowest power consumption and “set” voltage (0.2 V) among the perovskite-based memristors without double metal electrodes or additional insulating layer. Future explorations of 1D OIHP devices, which can combine the light-detection, resistive memory, and logic calculation, are highly expected. We are optimistic that by the simple fabrication and fascinating attributes of 1D OIHP materials, their electronic and optoelectronic devices could be pushed more for the development of flexible, low-power, and multifunctional applications.

## 4. Materials and Methods

### 4.1. Synthesis and Material Preparation

1D (IFA)_3_PbI_5_ crystals were synthesized by adding lead iodide (99.999%) powder (1.844 g, 4.0 mmol) into aqueous hydroiodic acid (57% by weight, 24 mL) at 75°C. In a flowing argon atmosphere, 0.168 g (4.0 mmol) amount of cyanamide was added to the solution and kept stirring for 30 min. Then, the mixed solution was cooling to precipitate (IFA)_3_PbI_5_ crystals. (IFA)_3_PbI_5_ solution was prepared by magnetic stirring at room temperature for 2 h by dissolving (IFA)_3_PbI_5_ (168 mg) in a dual solvent of dimethyl sulfoxide (DMSO) and N,N-dimethylformamide (DMF, 160 *μ*L). Keeping DFM unchanged at 160 *μ*L, we configured solutions with four concentrations, in which the DMSO volumes were 8.5 *μ*L, 17 *μ*L, 25.5 *μ*L, 34 *μ*L, and 42.5 *μ*L, corresponding to the molar ratios of (IFA)_3_PbI_5_ to DMSO of 1 : 1, 1 : 2, 1 : 3, 1 : 4, and 1 : 5, respectively.

### 4.2. Device Fabrication

Indium tin oxide- (ITO-) coated glass substrates were sequentially cleaned in acetone, isopropanol, and ethanol for 15 min and then dried using nitrogen gas. The clean substrates were treated in UV-ozone for 30 min before being transformed into a nitrogen-filled glove box for device fabrication. To obtain highly uniform and smooth (IFA)_3_PbI_5_ film, the preprepared solution was spin-coated on the ITO surface with a spin-coating rate of 3000 rpm for 30 s. Then, the chlorobenzene was quickly dropped onto the center of the substrate during spin-coating. Subsequently, the film was annealed on a hot plate at 100°C for 5 min. Then, (IFA)_3_PbI_5_ film was cooled to room temperature. To complete the device fabrication, the top metal electrodes (Au, 60 nm) were deposited on the (IFA)_3_PbI_5_ film by thermal evaporation at pressure of $5\times 10^{-4}$ Torr using a shadow mask.

### 4.3. Characterization

The crystal structure was characterized by X-ray diffraction (XRD) with Cu K*α* radiation (Bruker AXS D8) and X-ray single crystal diffractometer (BRUKER D8 QUEST). The decomposition temperature was measured using the Thermal Gravimetric Analyzer (METTLER TOLEDO TGA2). Bandgap was confirmed by absorbance spectra using a UV–vis spectrophotometer (SHIMADZU UV-1750). Atomic force microscope (AFM, Park XE-70) and scanning electron microscope (SEM, JEOL JSM-7800F) were used to characterize the morphologies and uniformity. The thickness of (IFA)_3_PbI_5_ was measured by step profiler (KLA Tencor). Typical current-voltage (*I-V*) characteristic curves of memory cells were measured in the atmosphere using a semiconductor parameter analyzer (Keithley 4200-SCS).

## Data Availability

All data needed to evaluate the conclusions in the paper are presented in the paper and/or the Supplementary Materials. Additional data related to this paper may be requested from the authors.

## Supplementary Materials

Supplementary Materials Table S1: single crystal data of 1D (IFA)_3_PbI_5_. Fig. S1: surface morphology and resistive switching property of 1D (IFA)_3_PbI_5_ prepared with a molar ratio of (IFA)_3_PbI_5_ to DMSO of 1 : 5. Fig. S2: surface morphology of 1D (IFA)_3_PbI_5_.

## References

[1] R. J. Sutton, G. E. Eperon, L. Miranda, E. S. Parrott, B. A. Kamino, J. B. Patel, M. T. Horantner, M. B. Johnston, A. A. Haghighirad, D. T. Moore, and H. J. Snaith, “Bandgap-tunable cesium lead halide perovskites with high thermal stability for efficient solar cells,” *Advanced Energy Materials*, vol. 6, no. 8, p. 1502458, 2016

[2] Y. Wang, T.-S. Su, H.-Y. Tsai, T.-C. Wei, and Y. Chi, “Spiro-phenylpyrazole/fluorene as hole-transporting material for perovskite solar cells,” *Scientific Reports*, vol. 7, no. 1, p. 7859, 2017 28798387 10.1038/s41598-017-08187-4 PMC5552831

[3] D. Shi, V. Adinolfi, R. Comin, M. Yuan, E. Alarousu, A. Buin, Y. Chen, S. Hoogland, A. Rothenberger, K. Katsiev, Y. Losovyj, X. Zhang, P. A. Dowben, O. F. Mohammed, E. H. Sargent, and O. M. Bakr, “Low trap-state density and long carrier diffusion in organolead trihalide perovskite single crystals,” *Science*, vol. 347, no. 6221, pp. 519–522, 2015 25635092 10.1126/science.aaa2725

[4] W. Li, Z. Wang, F. Deschler, S. Gao, R. H. Friend, and A. K. Cheetham, “Chemically diverse and multifunctional hybrid organic–inorganic perovskites,” *Nature Reviews Materials*, vol. 2, no. 3, p. 16099, 2017

[5] T. Baikie, Y. Fang, J. M. Kadro, M. Schreyer, F. Wei, S. G. Mhaisalkar, M. Graetzel, and T. J. White, “Synthesis and crystal chemistry of the hybrid perovskite (CH3NH3)PbI3 for solid-state sensitised solar cell applications,” *Journal of Materials Chemistry A*, vol. 1, no. 18, pp. 5628–5641, 2013

[6] P. Gao, M. Graetzel, and M. K. Nazeeruddin, “Organohalide lead perovskites for photovoltaic applications,” *Energy & Environmental Science*, vol. 7, no. 8, pp. 2448–2463, 2014

[7] F. Wang, Q. Chang, Y. Yun, S. Liu, Y. Liu, J. Wang, Y. Fang, Z. Cheng, S. Feng, L. Yang, Y. Yang, W. Huang, and T. Qin, “Hole-Transporting Low-Dimensional Perovskite for Enhancing Photovoltaic Performance,” *Research*, vol. 2021, article 9797053, pp. 1–11, 2021 10.34133/2021/9797053 PMC8328399 34386771

[8] X. Chang, Y. Fan, K. Zhao, J. Fang, D. Liu, M.-C. Tang, D. Barrit, D.-M. Smilgies, R. Li, J. Lu, J. Li, T. Yang, A. Amassian, Z. Ding, Y. Chen, S. Liu, and W. Huang, “Perovskite solar cells toward eco-friendly printing,” *Research*, vol. 2021, article 9671892, p. 11, 2021 10.34133/2021/9671892 PMC7906024 33681813

[9] Z. Xiao, R. A. Kerner, L. Zhao, N. L. Tran, K. M. Lee, T.-W. Koh, G. D. Scholes, and B. P. Rand, “Efficient perovskite light-emitting diodes featuring nanometre-sized crystallites,” *Nature Photonics*, vol. 11, no. 2, pp. 108–115, 2017

[10] Y. Sun, L. Zhang, N. Wang, S. Zhang, Y. Cao, Y. Miao, M. Xu, H. Zhang, H. Li, C. Yi, J. Wang, and W. Huang, “The formation of perovskite multiple quantum well structures for high performance light-emitting diodes,” *NPJ Flexible Electronics*, vol. 2, no. 1, p. 12, 2018

[11] L. Cheng, C. Yi, Y. Tong, L. Zhu, G. Kusch, X. Wang, X. Wang, T. Jiang, H. Zhang, J. Zhang, C. Xue, H. Chen, W. Xu, D. Liu, R. A. Oliver, R. H. Friend, L. Zhang, N. Wang, W. Huang, and J. Wang, “Halide homogenization for high-performance blue perovskite electroluminescence,” *Research*, vol. 2020, article 9017871, p. 10, 2020 10.34133/2020/9017871 PMC7877380 33623912

[12] A. K. Jena, A. Kulkarni, and T. Miyasaka, “Halide perovskite photovoltaics: background, status, and future prospects,” *Chemical Reviews*, vol. 119, no. 5, pp. 3036–3103, 2019 30821144 10.1021/acs.chemrev.8b00539

[13] L. Cheng, Y. Li, K. S. Yin, S. Y. Hu, Y. T. Su, M. M. Jin, Z. R. Wang, T. C. Chang, and X. S. Miao, “Functional demonstration of a memristive arithmetic logic unit (MemALU) for in‐memory computing,” *Advanced Functional Materials*, vol. 29, no. 49, p. 1905660, 2019

[14] J.-Y. Mao, Z. Zheng, Z.-Y. Xiong, P. Huang, G.-L. Ding, R. Wang, Z.-P. Wang, J.-Q. Yang, Y. Zhou, T. Zhai, and S.-T. Han, “Lead-free monocrystalline perovskite resistive switching device for temporal information processing,” *Nano Energy*, vol. 71, p. 104616, 2020

[15] J. Choi, S. Park, J. Lee, K. Hong, D.-H. Kim, C. W. Moon, G. D. Park, J. Suh, J. Hwang, S. Y. Kim, H. S. Jung, N.-G. Park, S. Han, K. T. Nam, and H. W. Jang, “Organolead halide perovskites for low operating voltage multilevel resistive switching,” *Advanced Materials*, vol. 28, no. 31, pp. 6562–6567, 2016 27192161 10.1002/adma.201600859

[16] E. J. Yoo, M. Lyu, J.-H. Yun, C. J. Kang, Y. J. Choi, and L. Wang, “Resistive switching behavior in organic-inorganic hybrid CH_3_NH_3_PbI_3−x_Cl_x_ perovskite for resistive random access memory devices,” *Advanced Materials*, vol. 27, no. 40, pp. 6170–6175, 2015 26331363 10.1002/adma.201502889

[17] S. Gao, X. Yi, J. Shang, G. Liu, and R.-W. Li, “Organic and hybrid resistive switching materials and devices,” *Chemical Society Reviews*, vol. 48, no. 6, pp. 1531–1565, 2019 30398508 10.1039/c8cs00614h

[18] B. Hwang, and J. S. Lee, “A strategy to design high-density nanoscale devices utilizing vapor deposition of metal halide perovskite Materials,” *Advanced Materials*, vol. 29, no. 29, p. 1701048, 2017 10.1002/adma.201701048 28558134

[19] P. Noe, C. Vallee, F. Hippert, F. Fillot, and J.-Y. Raty, “Phase-change materials for non-volatile memory devices: from technological challenges to materials science issues,” *Semiconductor Science and Technology*, vol. 33, no. 1, article 013002, 2018

[20] X.-F. Cheng, X. Hou, J. Zhou, B.-J. Gao, J.-H. He, H. Li, Q.-F. Xu, N.-J. Li, D.-Y. Chen, and J.-M. Lu, “Pseudohalide-induced 2D (CH_3_NH_3_)_2_PbI_2_(SCN)_2_ perovskite for ternary resistive memory with high performance,” *Small*, vol. 14, no. 12, p. 1703667, 2018 10.1002/smll.201703667 29457377

[21] J. Zhang, X. Song, L. Wang, and W. Huang, “Ultrathin two-dimensional hybrid perovskites toward flexible electronics and optoelectronics,” *National Science Review*, no. article nwab129, 2021 10.1093/nsr/nwab129 PMC9113132 35591916

[22] B. Hwang, C. Gu, D. Lee, and J. S. Lee, “Effect of halide-mixing on the switching behaviors of organic-inorganic hybrid perovskite memory,” *Scientific Reports*, vol. 7, no. 1, p. 43794, 2017 28272547 10.1038/srep43794 PMC5341555

[23] D. J. Liu, Q. Q. Lin, Z. G. Zang, M. Wang, P. H. Wangyang, X. S. Tang, M. Zhou, and W. Hu, “Flexible all-inorganic perovskite CsPbBr_3_ nonvolatile memory device,” *ACS Applied Materials & Interfaces*, vol. 9, no. 7, pp. 6171–6176, 2017 28112895 10.1021/acsami.6b15149

[24] K. Leng, W. Fu, Y. Liu, M. Chhowalla, and K. P. Loh, “From bulk to molecularly thin hybrid perovskites,” *Nature Reviews Materials*, vol. 5, no. 7, pp. 482–500, 2020

[25] A. Solanki, A. Guerrero, Q. Zhang, J. Bisquert, and T. C. Sum, “Interfacial mechanism for efficient resistive switching in Ruddlesden–Popper perovskites for non-volatile memories,” *Journal of Physical Chemistry Letters*, vol. 11, no. 2, pp. 463–470, 2020 31873017 10.1021/acs.jpclett.9b03181

[26] S. Y. Kim, J. M. Yang, E. S. Choi, and N. G. Park, “Layered (C_6_H_5_CH_2_NH_3_)_2_CuBr_4_ perovskite for multilevel storage resistive switching memory,” *Advanced Functional Materials*, vol. 30, no. 27, p. 2002653, 2020

[27] H. Kim, M.-J. Choi, J. M. Suh, J. S. Han, S. G. Kim, Q. V. Le, S. Y. Kim, and H. W. Jang, “Quasi-2D halide perovskites for resistive switching devices with on/off ratios above 109,” *NPG Asia Materials*, vol. 12, no. 1, p. 21, 2020

[28] J. Choi, J. S. Han, K. Hong, S. Y. Kim, and H. W. Jang, “Organic-inorganic hybrid halide perovskites for memories, transistors, and artificial synapses,” *Advanced Materials*, vol. 30, no. 42, p. 1704002, 2018 10.1002/adma.201704002 29847692

[29] Y.-K. Ren, S.-D. Liu, B. Duan, Y.-F. Xu, Z.-Q. Li, Y. Huang, L.-H. Hu, J. Zhu, and S.-Y. Dai, “Controllable intermediates by molecular self-assembly for optimizing the fabrication of large-grain perovskite films via one-step spin-coating,” *Journal of Alloys and Compounds*, vol. 705, pp. 205–210, 2017

[30] X. Xu, X. Zhang, W. Deng, J. Jie, and X. Zhang, “1D organic-inorganic hybrid perovskite micro/nanocrystals: fabrication, assembly, and optoelectronic applications,” *Small Methods*, vol. 2, no. 7, p. 1700340, 2018

[31] J.-M. Yang, S.-G. Kim, J.-Y. Seo, C. Cuhadar, D.-Y. Son, D. Lee, and N.-G. Park, “1D hexagonal HC(NH_2_)_2_PbI_3_ for multilevel resistive switching nonvolatile memory,” *Advanced Electronic Materials*, vol. 4, no. 9, p. 1800190, 2018

[32] C. Gu, and J.-S. Lee, “Flexible hybrid organic–inorganic perovskite memory,” *ACS Nano*, vol. 10, no. 5, pp. 5413–5418, 2016 27093096 10.1021/acsnano.6b01643

[33] F. Zhou, Y. Liu, X. Shen, M. Wang, F. Yuan, and Y. Chai, “Low-voltage, optoelectronic CH_3_NH_3_PbI_3−x_Cl_x_ memory with integrated sensing and logic operations,” *Advanced Functional Materials*, vol. 28, no. 15, p. 1800080, 2018

[34] C. Cuhadar, S.-G. Kim, J.-M. Yang, J.-Y. Seo, D. Lee, and N.-G. Park, “All-inorganic bismuth halide perovskite-like materials A_3_Bi_2_I_9_ and A_3_Bi_1.8_Na_0.2_I_8.6_ (A = Rb and Cs) for low-voltage switching resistive memory,” *ACS Applied Materials & Interfaces*, vol. 10, no. 35, pp. 29741–29749, 2018 29968458 10.1021/acsami.8b07103

[35] H. Ye, B. Sun, Z. Wang, Z. Liu, X. Zhang, X. Tan, T. Shi, Z. Tang, and G. Liao, “High performance flexible memristors based on a lead free AgBiI4perovskite with an ultralow operating voltage,” *Journal of Materials Chemistry C*, vol. 8, no. 40, pp. 14155–14163, 2020

[36] J. S. Han, Q. V. Le, J. Choi, K. Hong, C. W. Moon, T. L. Kim, H. Kim, S. Y. Kim, and H. W. Jang, “Air-atable cesium lead iodide perovskite for ultra-low operating voltage resistive switching,” *Advanced Functional Materials*, vol. 28, no. 5, p. 1705783, 2018

[37] Z. Xiong, W. Hu, Y. She, Q. Lin, L. Hu, X. Tang, and K. Sun, “Air-stable lead-free perovskite thin film based on CsBi_3_I_10_ and its application in resistive switching devices,” *ACS Applied Materials & Interfaces*, vol. 11, no. 33, pp. 30037–30044, 2019 31342747 10.1021/acsami.9b09080

[38] D. S. Jeong, R. Thomas, R. S. Katiyar, J. F. Scott, H. Kohlstedt, A. Petraru, and C. S. Hwang, “Emerging memories: resistive switching mechanisms and current status,” *Reports on Progress in Physics*, vol. 75, no. 7, article 076502, 2012 10.1088/0034-4885/75/7/076502 22790779

[39] Z. Xu, Z. Liu, Y. Huang, G. Zheng, Q. Chen, and H. Zhou, “To probe the performance of perovskite memory devices: defects property and hysteresis,” *Journal of Materials Chemistry C*, vol. 5, no. 23, pp. 5810–5817, 2017

[40] K. Yang, F. Li, C. P. Veeramalai, and T. Guo, “A facile synthesis of CH_3_NH_3_PbBr_3_ perovskite quantum dots and their application in flexible nonvolatile memory,” *Applied Physics Letters*, vol. 110, no. 8, article 083102, 2017

[41] X. Cao, Y. Han, J. Zhou, W. Zuo, X. Gao, L. Han, X. Pang, L. Zhang, Y. Liu, and S. Cao, “Enhanced switching ratio and long-term stability of flexible RRAM by anchoring polyvinylammonium on perovskite grains,” *ACS Applied Materials & Interfaces*, vol. 11, no. 39, pp. 35914–35923, 2019 31495172 10.1021/acsami.9b12931

[42] S. Meloni, T. Moehl, W. Tress, M. Franckevicius, M. Saliba, Y. H. Lee, P. Gao, M. K. Nazeeruddin, S. M. Zakeeruddin, U. Rothlisberger, and M. Graetzel, “Ionic polarization-induced current–voltage hysteresis in CH3NH3PbX3 perovskite solar cells,” *Nature Communications*, vol. 7, no. 1, p. 10334, 2016 10.1038/ncomms10334 PMC4748116 26852685

