# Supplementary material for: One-Dimensional (NH=CINH3)3PbI5 Perovskite for Ultralow Power Consumption Resistive Memory
Source: Research (Wash D C). 2021 Oct 8;2021:9760729. doi: 10.34133/2021/9760729 (PMC11014674; doi:10.34133/2021/9760729)
Supplement: Supplementary Materials — Table S1: single crystal data of 1D (IFA)3PbI5. Fig. S1: surface morphology and resistive switching property of 1D (IFA)3PbI5 prepared with a molar ratio of (IFA)3PbI5 to DMSO of 1 : 5. Fig. S2: surface morphology of 1D (IFA)3PbI5. [file 9760729.f1.docx]

**Supporting Information**

One-dimensional (NH=CINH_3_)_3_PbI_5_ Perovskite for Ultra-low Power Consumption Resistive Memory

Xuefen Song^1†^, Hao Yin^1†^, Qing Chang^1†^, Yuchi Qian^1^, Chongguang Lyu^1^, Huihua Min^2^, Xinrong Zong^1^, Chao Liu^1^, Yinyu Fang^1^, Zhengchun Cheng^1^, Tianshi Qin^1^*, Wei Huang^1,3^*, and Lin Wang^1^*

^1^Key Laboratory of Flexible Electronics (KLOFE) & Institute of Advanced Materials (IAM), Nanjing Tech University (Nanjing Tech), 30 South Puzhu Road, Nanjing 211816, China.

^2^Electron Microscope Laboratory, Nanjing Forestry University, Nanjing 210037, China.

^3^MIIT Key Laboratory of Flexible Electronics (KLoFE), Shaanxi Key Laboratory of Flexible Electronics (KLoFE), Xi'an Key Laboratory of Flexible Electronics (KLoFE), Xi'an Key Laboratory of Biomedical Materials & Engineering, Xi'an Institute of Flexible Electronics, Institute of Flexible Electronics (IFE), Northwestern Polytechnical University, Xi'an 710072, Shaanxi, China.

Correspondence should be addressed to Tianshi Qin; [iamtsqin@njtech.edu.cn](mailto:iamtsqin@njtech.edu.cn), Wei Huang; [iamwhuang@nwpu.edu.cn](mailto:iamwhuang@nwpu.edu.cn) and Lin Wang; [iamlwang@njtech.edu.cn](mailto:iamlwang@njtech.edu.cn).

† These authors contributed equally to this work.

***Table S1. Single crystal data of 1D (IFA)_3_PbI_5_.***

| **Formula** | **(NH=CINH_3_)_3_PbI_5_** | | |
| --- | --- | --- | --- |
| **Wavelength (Å)** | 0.71073 | | |
| **Cell Lengths (Å)** | a = 6.420(3) | b = 20.128(8) | c = 18.793(6) |
| **Cell Angles (°)** | α = 90.00 | β = 92.912(4) | γ = 90.00 |
| **Cell Volume (Å^3^)** | 2429.11(16) | | |
| **Temperature (K)** | 294.88 | | |
| **h_max_, k_max_, l_max_** | 7, 24, 22 | | |
| **h_min_, k_min_, l_min_** | -5,-23,22 | | |


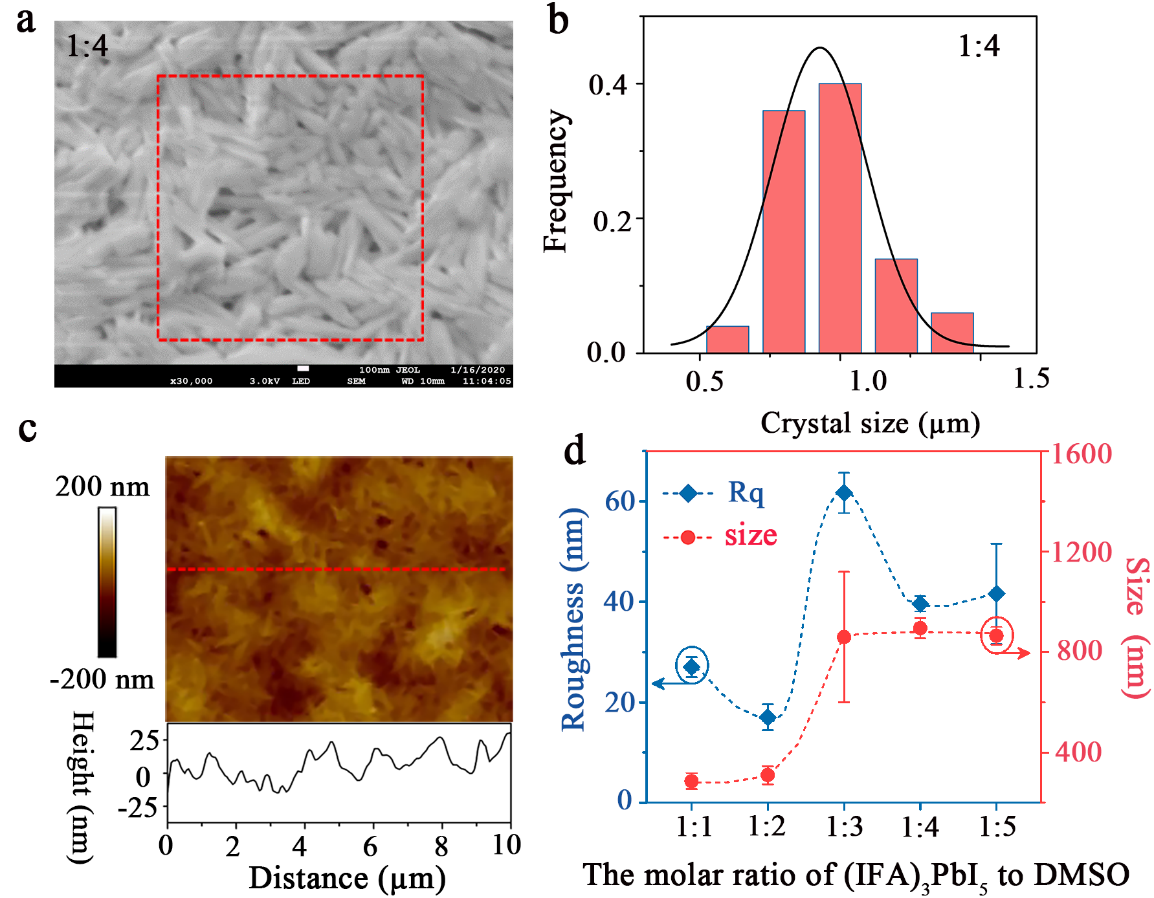


**Figure S1.** Surface morphology of 1D *(IFA)_3_PbI_5_.* (a) *SEM image* of a typical *film prepared with a molar ratio of (IFA)_3_PbI_5_ to DMSO of 1:4. Red dotted square corresponds to the statistical area of average size of 1D needle-like crystalline grains. (b) Histogram statistics of the average grain size using a professional software platform named as Nano Measurer. (c)* *Corresponding* AFM image and surface roughness (R_q_) distribution*. (d)* Surface roughness (R_q_) and *grain size are well controlled by the molar ratio of (IFA)_3_PbI_5_ to DMSO.*


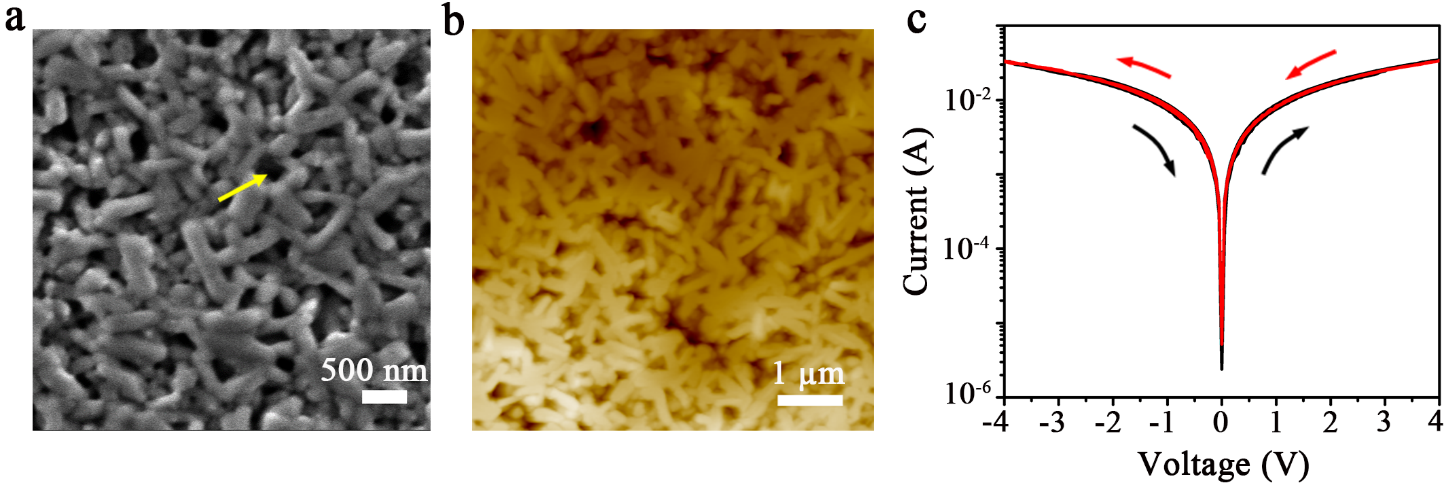


*Figure S2.* (a) AFM image and (b) SEM image *of 1D* (IFA)_3_PbI_5_ prepared *with a molar ratio of (IFA)_3_PbI_5_ to DMSO of 1:5. Obvious pinholes are pointed out by the yellow arrow. (c) Typical I-V curves of the first sweep of an Au/* (IFA)_3_PbI_5_ */ITO memory device fabricated by corresponding sample, maintaining at low resistance state without hysteresis behavior, perhaps caused by the large leakage current of the poor film.*
